# Subchondral Bone Remodeling: A Therapeutic Target for Osteoarthritis

**DOI:** 10.3389/fcell.2020.607764

**Published:** 2021-01-21

**Authors:** Xiaobo Zhu, Yau Tsz Chan, Patrick S. H. Yung, Rocky S. Tuan, Yangzi Jiang

**Affiliations:** ^1^Institute for Tissue Engineering and Regenerative Medicine, The Chinese University of Hong Kong, Hong Kong, China; ^2^Department of Orthopaedics & Traumatology, Faculty of Medicine, The Chinese University of Hong Kong, Hong Kong, China; ^3^School of Biomedical Sciences, The Chinese University of Hong Kong, Hong Kong, China

**Keywords:** osteoarthritis, subchondral bone, cellular and molecular targets, subchondral bone remodeling, regenerative therapy, stem cells

## Abstract

There is emerging awareness that subchondral bone remodeling plays an important role in the development of osteoarthritis (OA). This review presents recent investigations on the cellular and molecular mechanism of subchondral bone remodeling, and summarizes the current interventions and potential therapeutic targets related to OA subchondral bone remodeling. The first part of this review covers key cells and molecular mediators involved in subchondral bone remodeling (osteoclasts, osteoblasts, osteocytes, bone extracellular matrix, vascularization, nerve innervation, and related signaling pathways). The second part of this review describes candidate treatments for OA subchondral bone remodeling, including the use of bone-acting reagents and the application of regenerative therapies. Currently available clinical OA therapies and known responses in subchondral bone remodeling are summarized as a basis for the investigation of potential therapeutic mediators.

## Introduction

Osteoarthritis (OA) affects all tissues in diarthrodial joints, including articular cartilage, subchondral cortical bone, subchondral trabecular bone, and synovium, and the resultant pathological changes lead to pain, stiffness and dysfunction of the joint. Although articular cartilage loss and degeneration has long been considered the main cause of OA, and many therapies are designed to preserve articular cartilage, growing evidence suggests that the integrity and remodeling process of subchondral bone in OA joints, as an adaptation to cartilage degradation to maintain joint tissue homeostasis, also play an important role in OA physiopathology. Understanding the mechanism of subchondral bone remodeling in OA may provide insights for the design of future therapies to tackle OA at its early stage. This review will cover the structure and function of subchondral bone; describe the key regulatory factors and pathological changes of subchondral bone in OA joints; present the potential cellular and molecular therapeutic targets in subchondral bone remodeling and related current therapies, and possible strategies for future therapeutic design.

## Subchondral Bone in OA

### Structure and Function of Subchondral Bone

Articular cartilage is the hyaline connective tissue that covers the ends of bones in the diarthrodial joint, and it serves to absorb shock from joint movement. Articular cartilage has an aneural, avascular, and alymphatic structure composed of 65–80% water, 20–40% extracellular matrix (ECM), and 1–5% chondrocytes. The major components of ECM are collagens (mainly collagen type II) and proteoglycans (Carballo et al., 2017; Chen et al., 2017). Articular cartilage presents zonal structures from the articular surface (superficial, middle, and deep zones) to the conjunction area of subchondral bone (calcified zone), and the cell density and morphology differ in each zone (Jiang et al., 2017).

**Figure 1:**
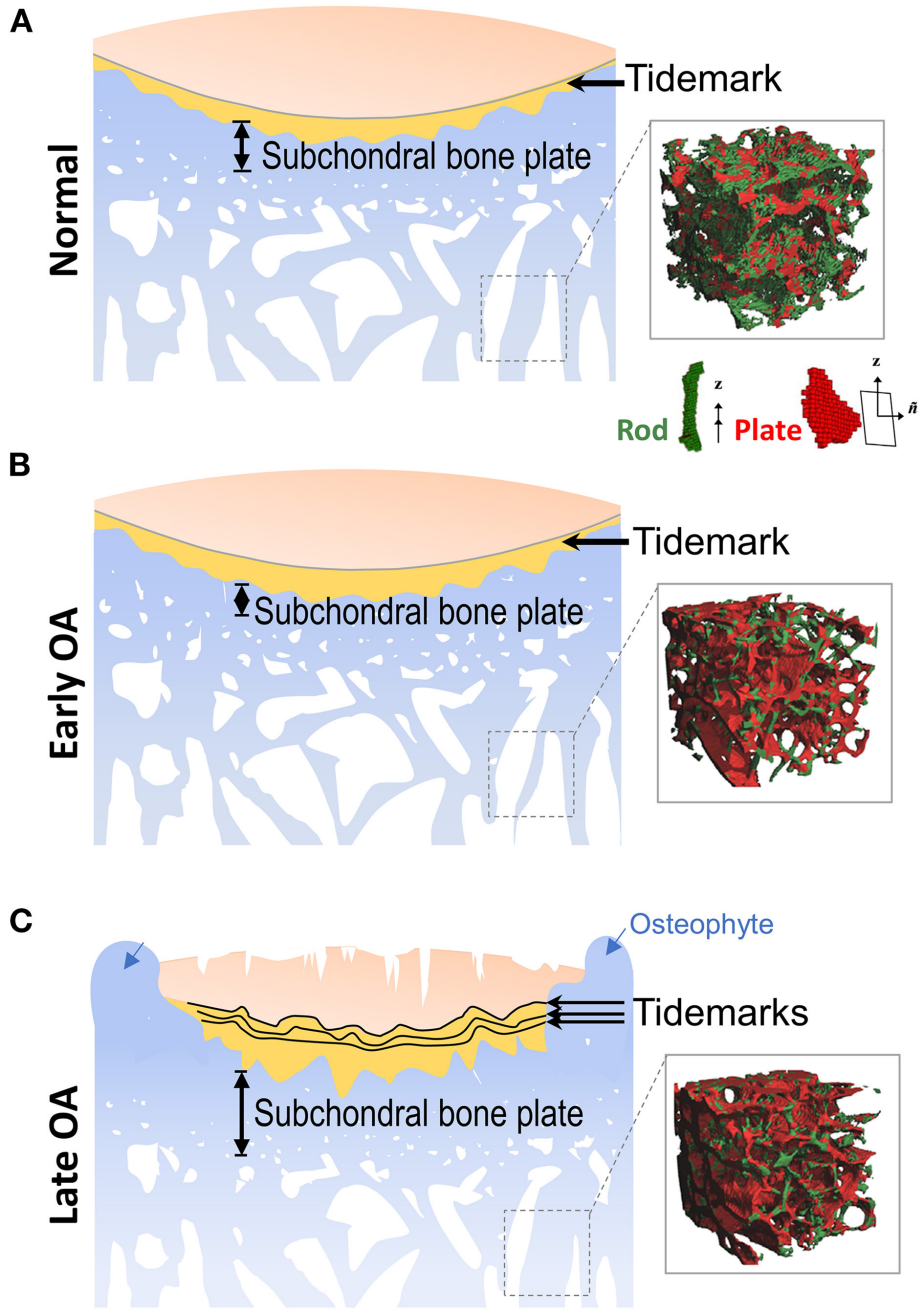
Alterations of subchondral bone during OA progression. **(A)** Located at the ends of bones, articular cartilage provides a low friction surface for weight bearing and joint movement, and is made up of four zones: superficial zone, middle zone, deep zone, and calcified zone. In the healthy joint, the thin layer of calcified zone/cartilage (yellow) present at the bottom of the articular cartilage is separated from the upper three zones by the histological marker termed tidemark. Subchondral bone is the bone tissue lying beneath the calcified cartilage, and includes both the subchondral cortical plate and subchondral trabecular bone, although there is no precise distinction for the differences between these two structures. (Inset) Depiction of the mineralized rod and plate structures in the trabecular bone. **(B)** In early OA, when the cartilage is still intact, the thickness of subchondral cortical plate is decreased due to elevated rate of bone remodeling. At the same time, bone loss also occurs in the subchondral trabecular bone. (Inset) A drastic loss of rod-like trabeculae and mild thickening of plate-like trabeculae is also detected in early OA. **(C)** In late OA, when degenerative changes are evident in articular cartilage, the thickness of the subchondral plate is increased, and the subchondral trabecular bone becomes sclerotic. Other periarticular bone changes, such as the formation of osteophytes (blue arrows) also occurs at this stage. The amount of calcified cartilage expands and penetrates into the upper hyaline articular cartilage, with the tidemark duplicated or disrupted. (Inset) The drastic loss of rod-like trabeculae and thickening of plate-like trabeculae continue in late OA. Inset figures depicting structure of trabecular plate and rod are reprinted with permission from Chen et al. (2018).

Subchondral bone refers to the bone tissue underlying the calcified cartilage and tidemark (Figure 1), including both subchondral cortical plate and subchondral trabecular bone. Subchondral bone plate is a thin layer of cortical bone lying immediately beneath the calcified cartilage. The physiological properties of the cortical plate are similar to the cortical bone located at other skeletal sites, providing mechanical strength to support the overlying articular cartilage, although the cortical plate is less stiff in comparison to diaphyseal cortical bone (Elastic modulus: 1.372 vs. 14 GN/m^2^) (Brown and Vrahas, 1984; Burr and Gallant, 2012). Underneath the cortical bone plate lies the subchondral trabecular bone, which is more porous and metabolically active compared to the cortical plate. The trabeculae in the cancellous bone have a unique structural network that adjusts to local mechanical influences (Figure 1A).

The osteochondral unit in the joint, which transfers load during joint movement, consists of articular cartilage, calcified cartilage and subchondral cortical and trabecular bone (Lories and Luyten, 2011; Goldring and Goldring, 2016). The normal subchondral cortical plate has a dense structure to support articular cartilage, and there are canals (10–160 mm in diameter) in the cortical plate, allowing the exchange of nutrients and molecules between cartilage and bone (Duncan et al., 1987; Clark, 1990; Bian et al., 2016).

**Figure 2:**
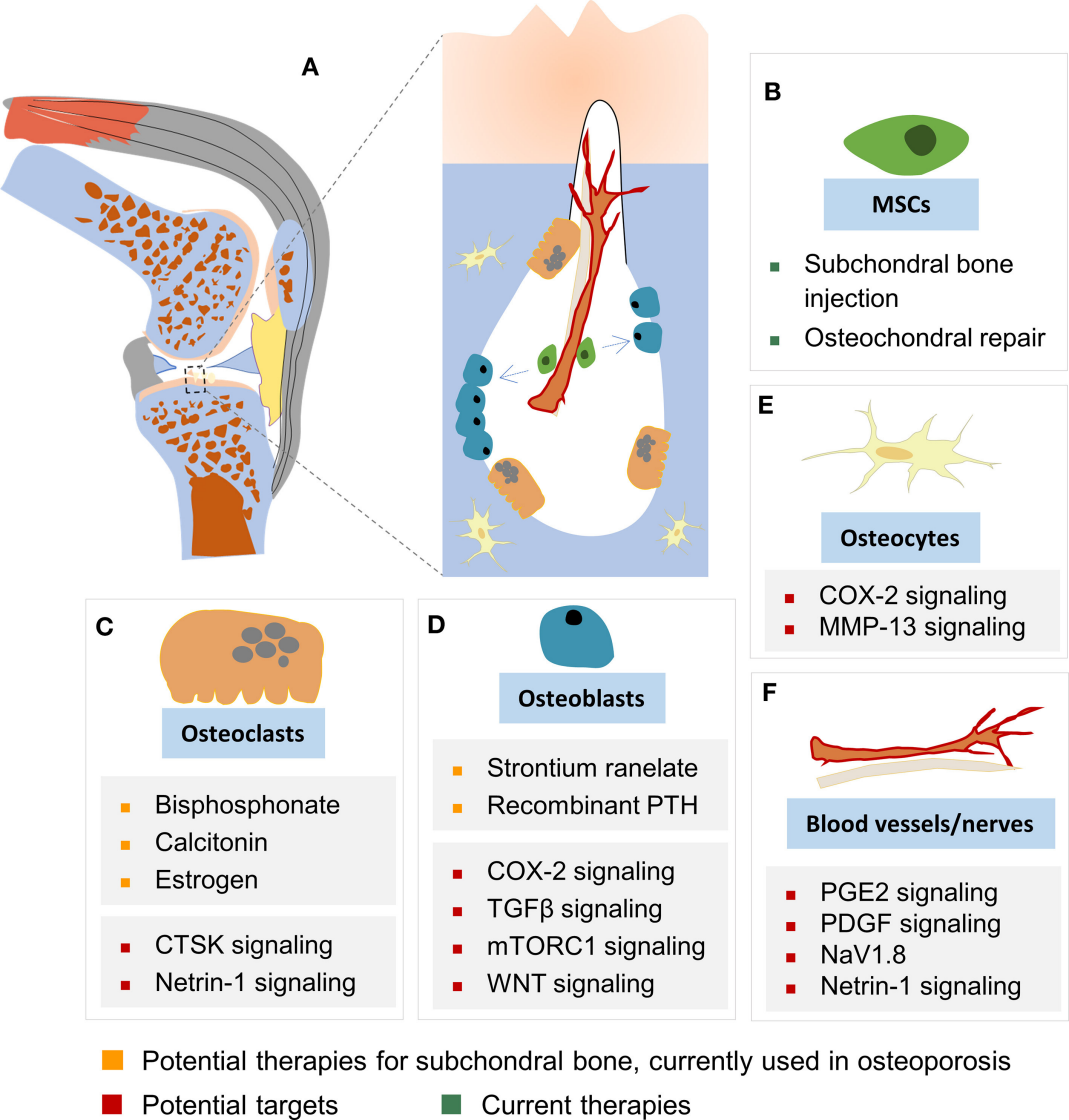
Cellular targets in subchondral bone remodeling. **(A)** Subchondral bone remodeling in OA (as illustrated in the knee). Cells and cellular structures depicted include MSCs **(B)**, osteoclasts **(C)**, osteoblasts **(D)**, osteocytes **(E)**, and blood vessels and nerves **(F)**. Increased osteoclast-mediated subchondral bone resorption (depicted by the large lacuna formed) at the onset of OA results in the release of growth factors previously embedded in bone ECM into the subchondral bone marrow. Subsequent actions of these factors, such as TGFβ contribute to angiogenesis, nerve innervation, and recruitment of MSCs and osteoprogenitors. These cellular processes together lead to activated bone formation, uncoupled bone remodeling, and disruption of the subchondral bone architecture. These alterations in the subchondral bone impair its mechanical properties of subchondral bone, such as load dissipation, and contribute to degeneration of articular cartilage. Current therapeutics, potential targets and potential therapies for subchondral bone remodeling are shown for: **(B)** MSCs, **(C)** osteoclasts, **(D)** osteoblasts, **(E)** osteocytes, and **(F)** blood vessels and nerves.

Articular cartilage stress distribution changes with subchondral bone expansion, and even a 1–2% incremental increase in the size of the subchondral bone could significantly increase stress to articular cartilage, as estimated by a recent human tibia data based computer-simulated model (Zhen et al., 2013). Upon injury or degeneration, the integrity of the osteochondral unit is breached, and the crosstalk between cartilage and subchondral bone is increased; the osteochondral unit is consequently altered and reshaped by the dynamic mechanical environment and biological microenvironment. Recently, Chen et al. (2020) reported a horizontal fissure at the osteochondral interface in obese OA patients, characterized by irregular cartilage erosion, fibro-granulation tissue infiltration, presence of free cartilage/bone debris and rupture of microcapillaries at the interface within the osteochondral unit. This represents a new type of OA pathological features, where neurovascular invasions were also identified in the degenerative osteochondral tissues (Li et al., 2013) (Figures 1B, 2A).

### Subchondral Bone in OA Joints

In OA joints, subchondral bone undergoes remarkable changes in both composition and structural organization, and has adverse effects on the overlying articular cartilage (Figure 1). With a bone scan (^99m^Tc pyrophosphate) and tetracycline labeling, Radin et al. (1984) provided early direct evidence that subchondral bone remodeling was significantly enhanced in response to mechanical loading in a rabbit model of hindlimb loading, and it preceded the fibrillation of the overlying articular cartilage (Radin et al., 1984). More histopathological features in osteoarthritic subchondral bone were then identified and discussed, including microdamage, bone marrow edema/lesions and bone cysts (Goldring and Goldring, 2016).

“Bone marrow edema” was termed and used about three decades ago, when Wilson et al. (1988) detected and localized regions of increased signal intensity in the subchondral bone of OA patients, with MRI based fluid-sensitive magnetic resonance sequences. However, histological examination of the subchondral bone in the anatomical edema sites revealed that local fat necrosis, marrow fibrosis and vascular changes were associated with microfractures of the trabecular bone, but not associated with edema (Leydet-Quilici et al., 2010). In current practice, people termed this pathological feature as bone marrow lesions (BMLs) in subchondral bone. Today, the different types and stages of BMLs can be identified by the radiological response to two MRI sequences, the fast spin-echo proton density (PDFS)-weighted and T1-weighted spin echo. Two major types of BMLs were designated based on the MRI readouts: BML1 represents an early-stage of the structural alteration in subchondral bone, which is detectable only by PDFS sequence, and BML2 is considered as a relatively more degenerative stage in the lesion sites, which is detectable by both PDFS and T1 sequence (Muratovic et al., 2016). Therefore, it appears that BMLs are correlated to the severity of OA progression (Taljanovic et al., 2008; Tanamas et al., 2010).

Subchondral bone cyst, also known as intra-osseous lesion, is another pathological feature often found in the load-bearing regions of the arthritic joints, but the exact pathogenesis of cyst formation is still unclear. Mechanical instability and overload may be the major cause. McErlain et al. (2012) created mechanically unstable OA models in rat knees by anterior cruciate ligament transection (ACLT) and partial medial meniscectomy (MMX); 75% of the knees developed subchondral bone cysts in 4 weeks, and all knees had subchondral bone cysts in 12 weeks. Moreover, enhanced bone turnover was also identified in human OA subchondral bone. Chen et al. (2015) examined cellular composition of human OA subchondral bone cysts and showed that the number of osteoclasts, osteoprogenitors (osterix^+^), osteoblasts (osteocalcin^+^, OCN^+^), and SOX9^+^ cells were higher in bone cyst sites than those in peri-cyst sites, suggesting enhanced bone turnover and abnormal bone remodeling.

Recently, the fine microarchitecture alterations of OA subchondral trabecular bone have been imaged and analyzed by advanced high-resolution micro-CT and MRI imaging systems. Liu et al. (2008) examined 71 human trabecular bone samples, and found that healthy trabecular bone consists of a network of rod-like and plate-like trabeculae microstructure (Figure 1A, right panel). Healthy subchondral trabecular bone displayed an even distribution of rod-like and plate-like trabeculae, and in human OA tibial plateau, a dramatic loss of rod-like trabeculae and thickening of plate-like trabeculae were identified using a novel microstructural analysis method, based on individual trabecula segmentation (Figures 1B,C, right panels) (Chen et al., 2018). Interestingly, in the same study, Chen et al. (2018) discovered that subchondral trabecular bone underneath the intact cartilage shared a similar phenotype with that underneath the severely damaged cartilage, which implied that the alterations of subchondral bone in early OA could precede cartilage degeneration.

## Subchondral Bone Remodeling in OA

Alterations in OA subchondral bone represent their adaptation to the changes of local biomechanical and biological signals, and are mediated by different types of bone cells (Goldring, 2009). These cell-regulatory adaptations conform to Wolff's law that the distribution and material properties of bone are dynamically determined by the applied load (Frost, 1994). Accordingly, when bone is subjected to increased loading, a number of bone properties change, including an expanding subchondral bone cross-sectional area, changes in bone mass, and remodeling of the trabeculae network (Teichtahl et al., 2015). Adult skeleton is constantly renewed during lifetime by bone remodeling, whereby mature bone tissue is removed from the skeleton (bone resorption) and new bone tissue is formed (bone formation). Bone remodeling controls the reshaping or replacement of bone following injuries, and this principle also applies to the subchondral bone, where increased bone resorption and alterations in its microarchitecture have been identified in both rheumatoid arthritis and OA (Li et al., 2014). Osteoclasts, mononuclear cells, osteoblasts, and osteocytes are the major cell types that participate in bone remodeling (Sims et al., 2015).

### Cells in Subchondral Bone Remodeling

#### Osteoclasts and Osteoclastogenesis in Subchondral Bone Resorption

Osteoclasts (tartrate-resistant acid phosphatase positive, TRAP^+^) are multinucleated cells that originate from bone marrow myeloid progenitor cells (TRAP^−^), and are the major cell type responsible for bone resorption (Teitelbaum, 2000; Katsimbri, 2017). During osteoclastogenesis, progenitor cells (TRAP^−^) are recruited to specific sites on bone surface, and differentiate to pre-osteoclast (TRAP^+^, mononucleated cells) and fuse to form multinucleated mature osteoclasts. Mature osteoclasts form a sealing zone on bone surface, and release hydrogen ions and catalytic enzymes for bone dissolution.

Osteoclasts are involved in arthritic bone diseases (Schett et al., 2005). Interestingly, peripheral blood mononuclear cells (PBMCs) from OA patients showed a higher level of osteoclastogenesis. Durand et al. (2013) isolated PBMCs from 140 OA patients, which were differentiated into osteoclasts for 3 weeks. OA-PBMCs generated more osteoclasts, and showed higher bone resorption rate when cultured on top of bovine cortical bone slides, compared to healthy controls (Durand et al., 2013). RANKL (receptor activator of nuclear factor kappa-B ligand, also known as osteoprotegerin ligand, osteoclast differentiation factor), exerts its function by binding to its receptor RANK on the surface of osteoclast precursors, and is one of the key regulators of osteoclastogenesis (Borciani et al., 2020). Increased osteoclast population was also observed in subchondral bone plates of OA patients (Jaiprakash et al., 2012).

The remodeling events in the subchondral bone in OA are not uniform across the joint because of different load-bearing conditions, and the remodeling rates may also vary with disease progression. For instance, active bone remodeling was found in bone cysts, but not in non-cyst areas, where the cyst cavity was created by activated osteoclasts, and bone formation was enhanced by osteoprogenitors (Osterix^+^) and osteoblasts (OCN^+^) (Chen et al., 2015). The high rate of subchondral bone resorption in OA is probably caused by increased osteoclast population and activity, and decreased cell apoptosis in a resorption cycle (Jaiprakash et al., 2012).

One of the key enzymes expressed by osteoclasts for bone resorption is cathepsin K (CTSK). Ctsk knockout mice (Ctsk^−/−^) maintained cartilage volume and structure in OA surgical model of destabilization of the medial meniscus (DMM model), although the TRAP^+^ osteoclast population in subchondral bone was increased in both wild type and Ctsk^−/−^ mice (Soki et al., 2018), and the subchondral bone was also preserved in OA Ctsk^−/−^ mice (Soki et al., 2018).

#### Mononuclear Cells in Subchondral Bone Reversal Phase

There is a reversal phase in bone remodeling cycle between bone resorption and bone formation, and so far little is known about it (Jensen et al., 2014; Sims and Martin, 2020). Reversal phase is a step to prepare the bone surface for bone formation, including clearing away the resorption debris and stimulating MSC proliferation, lineage commitment toward pre-osteoblast, differentiation and migration. Mononuclear cells are observed on the newly-resorbed bone surface, and are believed as the key cellular players in reversal phase, thus termed as reversal cells. These mononuclear cells are thought to be originated from hemopoietic-lineage mononuclear phagocytes, and with the possibility from osteoblast-lineage cells (Tran Van et al., 1982; Delaisse, 2014; Sims et al., 2015). The exact role of reversal cells is still under investigation, however, it is possible that the reversal phase might be the key therapeutic window to regulate subchondral bone remodeling in OA.

#### Osteoblasts, Osteoblastogenesis, and Subchondral Bone Sclerosis

Osteoblasts are differentiated from mesenchymal cells, and undergo four maturational stages - preosteoblasts, osteoblasts, bone-lining cells, and osteocytes, and contribute to bone formation (Clarke, 2008; Katsimbri, 2017). In OA, osteoblast phenotype and cell activity shift in the subchondral bone. Alkaline phosphatase (ALP) activity, releasing of RANKL (Kwan Tat et al., 2008), OCN, transforming growth factor β1 (TGFβ1) (Abed et al., 2017), insulin-like growth factor 1 (IGF1) (Hilal et al., 1998), and vascular endothelial growth factor (VEGF) (Corrado et al., 2013) are elevated in OA subchondral bone osteoblasts compared to normal osteoblasts, and the elevation of biofactors also induces a cascade of down-stream events, including sclerosis (Wang et al., 2013), osteoclastogenesis, and angiogenesis.

One of the most pronounced alterations in OA subchondral bone is sclerosis, which is usually found in the late stage of OA. A sclerotic subchondral bone has several structural characteristics, including the increased bone volume and density, thickening of subchondral bone plate, increased trabecular thickness, and decreased trabecular separation (Figure 1C). The microarchitecture of sclerotic subchondral trabeculae is transformed from rod-like into more plate-like (Figure 1C, right panel) (Li et al., 2013). Importantly, although bone volume and density are increased in sclerotic subchondral bone, the degree of mineralization is inadequate. The material stiffness of OA subchondral bone is therefore decreased, which may exaggerate cartilage degeneration upon joint loading (Li and Aspden, 1997). One possible explanation for subchondral bone sclerosis is that OA subchondral bone is actively being remodeled, and there is active recruitment of bone marrow and subchondral bone-resident progenitor cells and their induction toward osteogenesis; however, new bone formation and maturation are significantly impaired by the OA microenvironment (Bianco et al., 2018). Specifically, the mineralization ability of osteoblasts is reduced in the OA sclerotic subchondral cortical plate and trabecular bone tissues (Sanchez et al., 2008). The exact pathological mechanism is still under investigation. One of the possible reason is that OA sclerotic subchondral bone osteoblasts produce abnormal collagen type I (COL1), and both an aberrant formation of collagen type I homotrimer and a higher ratio of COL1A1 to COL1A2 have been reported (Bailey et al., 2002; Couchourel et al., 2009). Hypomineralization has also been found in OA sclerotic subchondral bone. One of the underlying molecular mechanisms is the elevated expression level of TGFβ in human OA subchondral bone osteoblasts, which may induce the expression of dickkopf-2 (DKK2), an inhibitor of mineralization (Chan et al., 2011). Recently, Sanchez et al. (2018) compared the secretome from human osteoblasts isolated from non-sclerotic and sclerotic areas, and identified 12 proteins about normal osteoblast function and mineralization that were significantly reduced in the osteoblasts from sclerotic subchondral bone.

#### Osteocytes

The role of osteocytes in OA subchondral bone is incompletely understood. As terminally differentiated osteoblasts embedded in bone matrix, osteocytes function as one type of mechanosensing cells (Robling and Bonewald, 2020), *via* mechanoloading-sensitive expression of RANKL to regulate osteoclastogenesis (Tatsumi et al., 2007; Nakashima et al., 2011; Xiong et al., 2011). Recent studies have also revealed a critical role for osteocyte-intrinsic TGFβ signaling in remodeling their surrounding bone matrix, a process termed perilacunar/canalicular remodeling (PLR), by which osteocytes sense the mechanical alterations, and resorb surrounding bone matrix by producing matrix metalloproteinases (MMPs), cathepsin and other proteolytic enzymes dynamically (Dole et al., 2017). Breakdown of PLR in subchondral bone osteocytes could induce an OA-like phenotype, even in the non-injured knee (Mazur et al., 2019), suggesting the important role of osteocytes in subchondral bone.

### Bone Extracellular Matrix

Bone ECM is produced by osteoblasts, and compositionally consists of mineral, water, collagen and noncollagenous proteins and lipids; the proportion of each component varies depending on species, age and site (Boskey and Robey, 2013). The osteoid matrix located at the endosteum of the bone is the newly secreted unmineralized matrix which then undergoes mineralization during osteogenesis, and collagen type I serves as the scaffold for the deposition of hydroxyapatite crystals in osteoid mineralization. The mechanical properties of bone are a function of the hardness of the mineralized ECM and the flexibility of organic components of the ECM. Bone ECM is the body's reservoir of minerals, macromolecules, and bioactive factors. It is the primary source of calcium, phosphate, and magnesium ions for maintenance of general physiological functions, including the maintenance of bone homeostasis. Bioactive factors are released into the marrow microenvironment upon bone resorption, and subsequently contribute to the recruitment and differentiation of stem/progenitor cells, such as mesenchymal stem cells (MSCs), during bone formation (Crane and Cao, 2014). For instance, bone matrix-derived IGF1 could stimulate osteoblastic differentiation of MSCs by activating mammalian target of rapamycin (mTOR), thus maintaining proper bone microarchitecture and mass (Xian et al., 2012). TGFβs are also abundant in bone ECM (TGFβ1, 188 ng/g; TGFβ2, 14 ng/g) (Hering et al., 2001), and activation of the TGFβ signaling pathway has been confirmed in OA subchondral bone, resulting in recruitment of MSCs and enhancement of bone formation (Zhen et al., 2013). In this manner, the subchondral bone ECM also participates in the regulation of bone homeostasis during OA bone remodeling.

## Current and Potential Therapies for OA Subchondral Bone Remodeling

The subchondral cortical bone and subchondral cancellous bone respond differently at early and late stages of OA (Figure 1) (Burr and Gallant, 2012; Goldring and Goldring, 2016). Subchondral bone plate thickness decreased at the early stage of OA (Intema et al., 2010), and elevation of bone resorption markers are found in the progressive OA patients (Bettica et al., 2002; Bolbos et al., 2008), accompanied by cartilage degeneration and enhanced vascularization in the subchondral bone (Bellido et al., 2010). At late stage of OA, subchondral sclerosis was reported with a 15% increase in bone mineral density and up to 30% increase in bone volume, accompanied by reduced mineralization (Hannan et al., 1993; Arden et al., 1996; Sanchez et al., 2008). Although there is no specific therapeutic treatment designed for subchondral bone remodeling, several bone-acting reagents are being investigated and pursued for their potential in regulating subchondral bone in OA patients (Figure 2).

### Inhibiting Osteoclasts in Subchondral Bone

#### Bisphosphonates

Bisphosphonates (BPs) are the most commonly used prescription drug for prevention and treatment of osteoporosis. BP binds to bone calcium and is released from the acidified bone surface, which is taken up by osteoclasts, and inhibits osteoclast activity by inducing osteoclast apoptosis (Coxon et al., 2008; Rogers et al., 2011). BPs have the potential to be used as OA subchondral bone regulating agents. For example, alendronate and zoledronate were reported to relieve subchondral bone pathological changes, thus effectively protecting the articular cartilage in OA animal models (Zhu et al., 2013; Lampropoulou-Adamidou et al., 2014). Clinically, the therapeutic effect of BPs for OA patients is controversial (Laslett et al., 2012; Cai et al., 2020). One up-to-date meta-analysis of randomized controlled trials involving 3,013 patients showed that BPs did not significantly improve OA in pain and structural and functional aspects compared to placebo (Vaysbrot et al., 2018). However, BPs may be suitable for OA patients with high bone turnover (Vaysbrot et al., 2018). A recent report by Hayes et al. (2020) suggested an early preventive effect of BPs for a subpopulation of OA patients (Hayes et al., 2020). Hayes et al. (2020) identified 346 BP responsive end-users from 1,977 eligible women, and in this cluster of patients, administration of BPs showed a protective effect against radiographic knee OA progression. The administration of BPs was particularly effective as early prevention for non-overweight patients, but less effective for those with more advanced disease or with higher weight-bearing stress (Hayes et al., 2020). These results suggest that BPs have the potential to be used as a prevention intervention for some early OA patients by regulating subchondral bone remodeling. Apart from subchondral bone, certain type of BPs, such as chlodronate, was also shown to have a protective effect on cartilage, acting *via* a purinergic receptor pathway (Rosa et al., 2014).

#### Calcitonin

Calcitonin is a 32-amino acid hormone produced by parafollicular cells of the thyroid gland that can inhibit osteoclast activity in bone. Similar to BPs, calcitonin also shows the capacity to prevent the loss of subchondral trabecular bone in an animal model (Behets et al., 2004), and exhibits chondro-protective effect in animal OA models (Wen et al., 2016). However, a recent Phase III clinical trial reported that the administration of calcitonin did not provide reproducible improvements on joint space width and pain relief (Karsdal et al., 2015). Moreover, due to the risk of cancer with long-term use of calcitonin, the European Medicines Agency concluded that the benefits of calcitonin could not outweigh their risks in the treatment of osteoporosis (European Medicines Agency, 2020). The application of calcitonin in OA subchondral bone remodeling should be further investigated and assessed.

#### Estrogen

Estrogen is important for bone development and health, and it regulates bone homeostasis by inhibiting osteoclast activation and inducing osteoclast apoptosis. Although estrogen has not been specifically subjected to randomized clinical trial for OA, some promising results provide support that estrogen has the potential to regulate OA-associated subchondral bone remodeling. One cross-sectional clinical study reported that elderly women with estrogen administration had a lower prevalence of knee OA–related subchondral bone attrition and subchondral bone edema-like lesions than those without estrogen administration (Carbone et al., 2004). Another cross-sectional study involving 4,366 white women found that estrogen replacement therapy can result in a lower risk of hip OA (Nevitt et al., 1996). Therefore, estrogen, estrogenic compounds, and selective estrogen receptor modulators (SERMs) may also be suitable for OA prevention in the postmenopausal patient cluster. There are also additional animal studies supporting this concept. Raloxifene (RAL), a commonly used drug for postmenopausal osteoporosis, could inhibit subchondral bone resorption, improve subchondral bone micro-architecture, and retard patellofemoral joint OA progression in ovariectomized rats (Bei et al., 2020). Another anti-postmenopausal osteoporosis drug, Tibolone, was also evidenced to increase serum ALP level and attenuated the development of knee OA in ovariectomized rats (Yang et al., 2014).

### Regulating Osteogenesis

#### Strontium Ranelate

Strontium ranelate (SrR), a bone-acting agent that has long been used as an anti-osteoporosis drug, was recently tested in clinic as a potential disease-modifying OA drug (DMOAD) to target subchondral bone. Strontium is a chemical analog of calcium that binds to bone and can enhance pre-osteoblast maturation, stimulate OPG production in osteoblasts, and inhibit osteoclast formation by regulating RANKL (Atkins et al., 2009; Tat et al., 2011). Interestingly, it was also found that SrR could enhance the cartilage matrix production rate of human chondrocytes *in vitro* (Henrotin et al., 2001).

A Phase III large international study, the SEKOIA trial (ISRCTN41323372), consisting of 1,371 OA patients over 50 years old with joint space width 2.5–5 mm who were treated with 1 g/day (*n* = 558), 2 g/day (*n* = 566), or placebo (*n* = 559) of SrR, reported structural and beneficial effects in the 1 g and 2 g /day groups, and symptomatic benefits in the 2 g/day group at 3 years (Reginster et al., 2013). Further follow-up also indicated improvement in pain and physical functions (Bruyere et al., 2014). The significance of the findings from this trial is the direct support of the possibility to target subchondral bone as early OA intervention. The remaining concerns of this drug are its side effects on the cardiovascular system that limit its applications, and the unclear patient subpopulation profile to identify proper responders.

#### Recombinant Human Parathyroid Hormone, Teriparatide

Recombinant human parathyroid hormone (PTH or teriparatide) has anabolic effects on bone formation by acting on osteoblasts, and has been approved to treat osteoporosis. In addition, emerging evidence suggested that PTH and parathyroid hormone related peptide (PTHrP) could directly influence articular cartilage homeostasis and bone growth (Chang et al., 2009; Sampson et al., 2011). Currently, there is no completed randomized clinical trial for treating OA with teriparatide. A Phase II clinical trial is under way to study the chondroprotective effect of teriparatide on participants with knee OA (NCT03072147), and the results on subchondral bone will be very relevant.

### Osteocytes

No current therapy is available to specifically target osteocytes.

### Other Components: Vascularization, Innervation

No current therapy is available.

## Future Therapeutic Targets

### Therapeutic Target: Inhibiting Osteoclasts

#### Cathepsin K Inhibitor

CTSK is an osteolytic protease involved in bone resorption and cartilage degradation by degrading bone matrix proteins. In OA, as subchondral bone remodeling exhibits aberrantly activated bone resorption, the CTSK pathway has been considered a potential therapeutic target. Administration of the CTSK inhibitor, SB-553484, delayed OA progression in a canine model (Connor et al., 2009). Another novel selective CTSK inhibitor, MIV-711, was recently developed for OA treatment, and showed promising protection against subchondral bone loss (both plate and trabecular bone) and partial alleviation of cartilage deterioration in early OA in rabbit and canine OA models (Lindstrom et al., 2018). In a Phase II clinical trial of MIV-711, although there was no impact on pain relief, effective reduction of bone resorption and cartilage volume loss was observed (Conaghan et al., 2020). Odanacatib is the only candidate CTSK inhibitor for human use, which is supported by the results from Phase III clinical trials, and exhibits high therapeutic efficacy in postmenopausal osteoporosis patients (Mcclung et al., 2019). However, the pharmaceutical company has discontinued development of Odanacatib due to adverse cardio-cerebrovascular effects and other concerns (Mullard, 2016).

### Therapeutic Target: Regulating Osteoblasts

#### TGFβ

TGFβ signaling is one of the key pathways that regulate bone development and remodeling (Kegelman et al., 2020). A high level of TGFβ1 activation was found in both human and mouse OA subchondral bone, which led to subchondral bone abnormality, including increased number of osteoblastic cells, enhanced subchondral bone sclerosis, and angiogenesis. Inhibition of TGFβ1 pathway *via* blocking TGFβ1 receptor (SB-505124) or neutralizing TGFβ1 in subchondral bone with neutralizing antibody (1D11) attenuated OA progression and rescued both articular cartilage from degeneration and subchondral bone from sclerosis (Zhen et al., 2013). Interestingly, researchers from the same group also reported that halofuginone, a natural quinazolinone alkaloid found in the Chinese herb *Dichroa febrifuga*, showed similar TGFβ1 inhibition effects in OA mouse models (Cui et al., 2016), suggesting that halofuginone may have potential therapeutic effect on regulating subchondral bone in OA. Another Chinese herbal extract isolated from the plant *Artemisia annua*, Artesunate, also inhibited subchondral bone TGFβ/Smad signaling pathway and restored the coupled bone remodeling in an ACLT-induced mice OA model (Li et al., 2019) (Table 1).

**Table 1:** Potential targets of subchondral bone remodeling for OA under investigation.

**OA phase**	**Potential target**	**Possible drug**	**References**
Early	Estrogen/Estrogen receptor	Raloxifene; Tibolone	Yang et al., 2014; Bei et al., 2020
	TGFβ	Halofuginone; Artesunate	Cui et al., 2016; Li et al., 2019
	COX2/PEG2	NSAIDs	Massicotte et al., 2002; Tu et al., 2019
Early-Late	PDGF; VEGF	Bevacizumab	Nagai et al., 2014
	H-Type (CD31^high^Emcn^high^) vessel	Defactinib	Kusumbe et al., 2014; Hu et al., 2020
N/A	YAP	Verteporfin	Zhang et al., 2020

#### Wnt

Canonical signaling *via* β-catenin is involved in bone remodeling and regulates joint development and disease, including subchondral bone (Huang et al., 2018). Activation of Wnt/β-catenin signaling has been found in OA subchondral bone, and inhibition of Wnt in osteoblasts by overexpression of its natural antagonist Dkk1, decreased OA phenotype, suggesting Wnt signaling as a possible pharmacological target for subchondral bone remodeling in OA (Funck-Brentano et al., 2014).

#### mTORC1

A newly discovered subchondral bone regulator is the mechanistic target of rapamycin complex 1 (mTORC1), a crucial element of the mTOR pathway. mTORC1 is overactivated in pre-osteoblasts in OA subchondral bone in both human and mice models. Lin et al. (2019) demonstrated that mTORC1 activation in pre-osteoblasts resulted in aberrant subchondral bone formation, and specific inhibition of mTORC1 pathway in pre-osteoblast could alleviate subchondral bone sclerosis and cartilage deterioration. Pharmacologic inhibition of mTOR pathway could be a possible target option for early stage OA treatment by regulating subchondral bone remodeling (Chan et al., 2011; Lin et al., 2019).

#### Other Chinese Herbs

Cai et al. (2018) reported that Magnoflorine, an herbal extract from *Pachygone ovata*, stimulated osteoblast proliferation and mineralization *in vitro* (25 μg /ml), and attenuated the degeneration of cartilage in early stage OA in a guinea pig spontaneous OA model. The drug delivery method consisted of 50 ng purified magnoflorine mixed with 2 μl hyaluronic acid gel to achieve sustained drug release *in situ* (Cai et al., 2018).

### Therapeutic Target: Osteocytes

Apart from osteoblasts and osteoclasts, emerging evidence suggests that the matrix-embedded osteocytes also play essential roles in bone remodeling, particularly in arthritic subchondral bone. Tu et al. (2019) reported that osteocytes in subchondral bone expressed high level of cyclooxygenase-2 (COX-2) during the development of OA in a spontaneous OA model (STR/Ort mice), where there was no significant changes in the number of tartrate-resistant acid phosphatase (TRAP)-positive osteoclasts, supporting the importance of osteocytes in early OA subchondral bone remodeling. Specifically, in this STR/Ort mice OA model, the high level of COX-2 expression in osteocytes gave rise to abnormal subchondral bone formation, and accelerated cartilage degeneration at the onset of OA. Application of COX-2 inhibitors to animals at 12.5–25% of the clinical application dosage for 4 weeks resulted in normalization of the microstructure of subchondral bone and relief of cartilage degeneration in mice; a similar effect was also found in a rheumatoid arthritis mice model (TNF-α transgenic RA mice). This important piece of pre-clinical evidence suggested a treating window for bone remodeling during the development of arthritis, and the current clinical available COX-2 inhibitors could also manage the subchondral bone remodeling *via* regulating the osteocytes (Tu et al., 2019).

In addition, subchondral bone osteocyte-derived matrix metallopeptidase 13 (MMP13) was also identified as a regulator of articular cartilage health, by affecting PLR of osteocytes. Mazur et al. (2019) established a MMP13 knockout mouse model with a Cre recombinase driven dentin matrix acidic phosphoprotein 1 (DMP1) promoter to specifically ablate osteocyte MMP13, without affecting chondrocyte derived MMP13. The depletion of osteocyte MMP13 increased both cortical and subchondral bone mass, and suppressed PLR during OA development in animals. Results from this study provided insights on the important contribution of osteocyte derived MMP13 to PLR during OA development, and on the dynamic crosstalk between subchondral bone osteocytes and cartilage (Mazur et al., 2019).

### Other Therapeutic Targets: Vascularization and Nerve Innervation

The vasculature in bone is indispensable for skeletal development and homeostasis. Osteogenesis is coupled with angiogenesis during bone modeling and remodeling (Brandi and Collin-Osdoby, 2006; Kusumbe et al., 2014; Xie et al., 2014). The role of angiogenesis in subchondral bone has been studied recently with genetic mouse models. Su et al. (2020) used transgenic mice to prove that overexpression of platelet-derived growth factor-BB (PDGF-BB) in preosteoclast spontaneously recapitulated the OA-like phenotype in subchondral bone by enhancing both angiogenesis and subchondral bone remodeling; meanwhile, conditional PDGF-BB knockout could effectively prevent surgically-induced OA progression (Su et al., 2020). These results suggested that subchondral bone angiogenesis is one of the key processes in subchondral bone remodeling and may be considered as a potential target. Besides intrinsically inhibiting subchondral bone vascularization, exogenous drugs targeting subchondral bone angiogenesis have also been studied. For example, bevacizumab (a VEGF antibody), halofuginone (inhibiting phosphorylation of Smad2/3 and TGFβ) and defactinib (inhibitor of focal adhesion kinase, FAK) all showed promising beneficial effects against OA in animal models *via* inhibition of subchondral vascularization (Cui et al., 2016; Lu et al., 2018; Wu et al., 2020).

Nerves often grow along new blood vessels into subchondral bone structures that are normally not innervated, which is also a feature of OA pathogenesis (Figure 2A), and may be related to pain (Mapp and Walsh, 2012). Recently, in a study of the role of osteoclast-initiated subchondral bone remodeling in sensory innervation and OA pain, Zhu et al. (2019) showed that osteoclast-initiated aberrant subchondral bone remodeling induced sensory innervation *via* subchondral osteoclast-derived Netrin-1, resulting in enhanced subchondral bone sensory nerve growth. This sensory innervation and OA pain could be attenuated by either inhibiting subchondral osteoclasts (by means of gene knockout in DMP1-Rankl Flox mice or treatment with alendronate), or deleting Netrin1 in osteoclast-lineage cells (by means of knockout in Trap-Netrin1 flox mice) (Zhu et al., 2019). Our group has recently reported the role of NGF/TrkA signaling pathway in calcification of human healthy articular chondrocyte, representing another mechanism operating on the other side of the cartilage tidemark that regulates OA subchondral remodeling (Jiang and Tuan, 2019). Another osteoblast-secreted inflammatory mediator, prostaglandin E2 (PGE2), was also found to be increased in OA subchondral bone and sensory innervation. PGE2 activates an ion channel protein, Nav1.8, and inhibition of PEG2 production or Nav1.8 function with a selective Nav1.8 sodium channel blocker A-803467 could attenuate OA (Zhu et al., 2020). Taken together, these observations provide insights on the linkage between sensory innervation and OA subchondral bone remodeling (Zhu et al., 2020).

That angiogenesis/vascularization and nerve innervation appear to play functional roles in subchondral bone remodeling during OA progression suggests that they are potential therapeutic targets for the development of OA drugs (Figure 2F).

### Regenerative Therapy

Regenerative therapies, including stem cell-based therapies are actively investigated for OA treatment. There are Phase I/II clinical trials of using bone marrow derived MSCs/BMSCs (Gupta et al., 2016; Shapiro et al., 2017), adipose tissue derived MSCs/ADSCs (Song et al., 2018; Lu et al., 2019), and other allogeneically derived MSCs (Matas et al., 2019) to treat OA knees (Table 2). For instance, two Phase II clinical trials showed that intra-articular injections of human ADSCs (5 × 10^7^ cells) exhibited the improvement in both pain relief and cartilage volume (Song et al., 2018; Lu et al., 2019). However, the favorable results reported are primarily focused on pain relief and functional improvement but few tissue regeneration and subchondral bone reestablishment, and the application is with the strict condition of administrative DOSES (Donor, Origin tissue, Separation method, Exhibited cell characteristics associated with behavior, and Site of delivery), i.e., cell derivation, cell number, harvesting method, sites of delivery (Murray et al., 2019) and frequency (Kim et al., 2019).

**Table 2:** Stem cell-based therapy for cartilage/osteochondral defect and OA.

**Disease condition**	**Cell sources**	**Sample size and group**	**Placebo/Control**	**Follow-up**	**Route of administration**	**Main results**	**Study group**
Cartilage defect	Autologous Chondrocytes	23 patients (13 femoral condyle defect due to trauma; 3 osteochondritis dissecans; 6 chondromalacia patellae; 1 traumatic origin)	N/A	39 months (16–66 months)	Autologous chondrocyte implantation	Transplants eliminated knee locking and reduced pain and swelling after surgery. Two-year follow-up showed that 14 (14/16) patients with femoral condylar transplants had good-to-excellent results. Three-year follow-up showed that 2 (2/7) patients with patellar transplants had excellent or good results.	Brittberg et al., 1994
		224 patients	N/A	12.8 years (10–20 year)		92% of the patients were satisfied with the treatment effect, and 74% of the patients reported same or better status as previous years. Improved clinical outcome, better life quality, and high activity level were reported.	Peterson et al., 2010
	CDPCs	15 patients	N/A	12 months	MACI, mini-arthrotomy	The cartilage defects (6–13 cm^2^) were completely repaired. Knee function (IKDC scores and Lysholm scores) was significantly improved.	Jiang et al., 2016
	BMSCs	24 patients; BMSCs (*n* = 12), Placebo (*n* = 12)	Cell-free collagen gel sheet	10.5 months (28–95 weeks)	Implantation at the time of HTO	Arthroscopic and histological grading scores were significantly improved, but no significant clinical improvement.	Wakitani et al., 2002
		3 patients	N/A	27 months	Transplantation surgery	The cartilage defects were repaired, and clinical symptoms were improved.	Wakitani et al., 2007
		1 patient	N/A	12 months	Arthroscopic surgery	The defect was recovered with hyaline-like cartilage tissue, and clinical symptoms were improved.	Kuroda et al., 2007
	SMSCs	10 patients (Symptomatic single cartilage lesion in femoral condyle)	N/A	52 months (37–80 months)	Arthroscopic surgery	The MRI score and Lysholm score were significantly improved compared to pre-surgery.	Sekiya et al., 2015
		5 patients (Symptomatic knee chondral lesions; 1.5-3.0 cm^2^)	N/A	24 months	Mini-arthrotomy	The defects were successfully repaired. And the VAS, Lysholm, and KOOS scores were significantly improved compared to pre-surgery.	Shimomura et al., 2018
Osteoarthritis	Bone marrow aspirate concentrate (BMAC)	25 patients (bilateral knee OA); BMACs into one knee joint and placebo into the contralateral knee	Sterile saline	6 months	Intra-articular injection	The pain relief (VAS scores and ICOAP scores) was significant compared to the baseline, but was non-significant compared to the saline treated contralateral knee.	Shapiro et al., 2017; Phase I
	BMSCs	60 patients; Cohort1: BMSCs dose1(2.5 × 10^7^ cells; *n* = 10), Placebo (*n* = 5); Cohort2: BMSCs dose2 (5 × 10^7^ cells; *n* = 10), Placebo (*n* = 5); Cohort3: BMSCs dose3 (7.5 × 10^7^ cells; *n* = 10), Placebo (*n* = 5); Cohort4: BMSCs dose4 (1.5 × 10^8^ cells; *n* = 10), Placebo (*n* = 5)	PLASMA-LYTE A (a multiple electrolytes injection)	12 months	Intra-articular injection	The lowest dose cohort (Cohort1; 2.5 × 10^7^ cells) showed a trend toward improvement in all parameters including VAS, WOMAC and ICOAP cores (though non-significant compared to the placebo group), while other higher doses cohorts neither showed significant symptomatic relief, nor showed acceptable safety.	Gupta et al., 2016; Phase II
	ADSCs	52 patients; ADSCs group (*n* = 26); Placebo (*n* = 26)	1% sodium Hyaluronic acid	12 months	Intra-articular injection	ADSCs group showed significant improvement in joint (WOMAC scores) and cartilage volume (MRI quantification) in comparison with both baseline and placebo group.	Lu et al., 2019; Phase IIb
		18 patients; ADSCs low-dose: (1 × 10^7^ cells; *n* = 6); ADSCs mid-dose: (2 × 10^7^ cells; *n* = 6); ADSCs high-dose: (5 × 10^7^ cells; *n* = 6)	N/A	24 months	Intra-articular injection	ADSCs group with different dosage all showed significant improvement in joint function (WOMAC scores), life quality (SF-36 scores) and cartilage volume (MRI quantification). The high dose group (5 × 10^7^ cells) exhibited the highest improvement.	Song et al., 2018; Phase I/II
		24 patients; ADSCs group (1 × 10^8^ cells; *n* = 12); Placebo (*n* = 12)	Sterile saline	6 months	Intra-articular injection	ADSCs group showed a significant functional improvement and pain relief in comparison with baseline, without cartilage volume changes at 6 months' follow-up.	Lee et al., 2019; Phase IIb
		30 patients; ADSCs single-injection group: (1 × 10^8^ cells; *n* = 10); ADSCs twice-injection group: (1 × 10^8^ cells, injection at baseline and 6 months; *n* = 10); Control group: (*n* = 10)	Conservative management	12 months	Intra-articular injection	The pain, function, and life quality of both treatment groups were significantly improved compared to the control group.	Freitag et al., 2019; Phase II
	UC-MSCs	26 patients; Single-dose UC-MSCs (20 × 10^6^ cells, *n* = 9); Repeated-dose UC-MSCs (20 × 10^6^ cells, twice a year, *n* = 9); Placebo (*n* = 8)	Hyaluronic acid	12 months	Intra-articular injection	Pain (WOMAC-A, pain scores) and function (WOMAC-C, function scores) were significantly improved in comparison with placebo group. Repeated-dose UC-MSCs (20 × 10^6^cells, twice a year) exhibited a superior effect to placebo group and single-dose group.	Matas et al., 2019; Phase I/II
		6 patients (KL grade 3 and ICRS cartilage defect grade 4); Low-dose group (1.15–1.25 × 10^7^cells; *n* = 3) High-dose group (1.65–2.0 × 10^7^cells; *n* = 3)	N/A	7 years	Intra-articular injection	The improved clinical outcomes were stable over 7 years of follow-up. The histological findings at 1 year showed hyaline-like cartilage. MRI at 3 years showed persistence of the regenerated cartilage. No cases of osteogenesis or tumorigenesis was observed over 7 years.	Park et al., 2017; Phase I/II

*ADSCs, adipose-derived mesenchymal stromal cells; BMSCs, Bone marrow-derived mesenchymal stromal cells; CDPCs, Cartilage derived progenitor cells; HTO, High tibial osteotomy; ICOAP, intermittent and constant osteoarthritis pain; ICRS, International Cartilage Repair Society; IKDC, International Knee Documentation Committee; KL, Kellgren-Lawrence; KOOS, Knee Injury and Osteoarthritis Outcome Score; MACI, matrix induced autologous chondrocyte implantation; MRI, magnetic resonance imaging; SMSCs, synovium-derived mesenchymal stromal cells; UC-MSCs, umbilical cord-derived mesenchymal stromal cells; VAS, visual analog scale; WOMAC, Western Ontario and McMaster Universities Arthritis Index*.

Regenerative therapy also represents an emerging strategy to enhance the subchondral bone health, particularly at the early stage of disease that could prevent OA progression. Subchondroplasty is a recently developed, clinically-used surgical treatment using bioactive materials, e.g., calcium phosphate, as bone substitute materials (BSMs), to fill the subchondral bone marrow lesions. The BSMs used for subchondroplasty are injectable, and are engineered products that have similar mechanical properties as the surrounding trabecular bone, thereby providing a bioactive scaffold for local subchondral bone remodeling, and preventing further damage to the osteochondral tissue (Farr and Cohen, 2013; Colon et al., 2015). Another approach is to use cell-based therapy to regenerate the osteochondral defects before it progresses to OA. Autologous chondrocytes (Brittberg et al., 1994; Peterson et al., 2010) and adult stem cells such as cartilage derived-progenitors (Jiang et al., 2016), bone marrow derived-MSCs (Wakitani et al., 2002, 2007; Kuroda et al., 2007) are the most frequently used cell types with satisfactory tissue repair results in clinical studies and applications (Jiang et al., 2017) (Table 2).

Tissue engineering constitutes a major strategy in regenerative therapy, i.e., to repair the tissue with exogenous cells, biomaterials, and/or bioactive factors. Another strategy is to enhance and push the limit of a tissue's self-healing capacity. Subchondral bone has a certain degree of self-repair ability in healthy joints. This healing capacity has been reported in different animal models, dependent on age and species (Pfeifer et al., 2017), size and site of the defect (Fisher et al., 2015; Orth et al., 2016), and is the foundation of the well-established surgical technique of microfracture. Microfracture has been used to treat small to medium sized cartilage/osteochondral defects by creating perpendicular channels into the subchondral bone plate and allowing the bone marrow to seep into the defect area (Pan et al., 2009). The cartilage repair/resurfacing resulting from this type of marrow stimulation techniques is thought to be partially due to the recruitment and local differentiation of bone marrow-derived MSCs, which act as a key player in subchondral bone remodeling and osteochondral homeostasis. We have previously established the application of a bioactive scaffold that achieved fast healing of subchondral bone in a rabbit osteochondral defect model. Interestingly, articular cartilage resurfacing was observed in the fast healing subchondral bone groups without any other additional treatments (Jiang et al., 2013). A subchondral remodeling step thus appears to take place in advance of cartilage repair in osteochondral healing, and the quality of cartilage generated from the marrow stimulation techniques is dependent on the healing speed and quality of subchondral bone remodeling (Chen et al., 2011; Jiang et al., 2013).

In this manner, a practical regenerative approach for OA is to enhance the performance of the subchondral bone by introducing additional bone marrow derived MSCs, to maintain osteochondral tissue homeostasis and prevent further cartilage loss. Some exciting results from a series of clinical trials have just been released by Hernigou et al. (2018, 2020a,b) (Table 3), with clinical data of up to 15 years of follow-ups. Patients with different OA conditions (moderate to advanced OA, 60 cases; advanced OA, 140 cases; OA secondary to knee osteonecrosis related to corticosteroids, 30 cases) were recruited. These patients have comparable clinical symptom in bilateral knees, and were randomized to accept subchondral bone MSC therapy or other surgical treatments. The subchondral bone MSC therapy was delivered to one knee, while the other knee was treated with either intraarticular MSC injection (*n* = 60) or total knee arthroplasty (TKA, *n* = 140) (Table 3, Study 1 and Study 2). These results showed that: (i) precise delivery of MSCs to subchondral bone could postpone TKA in OA patients for more than 10 years; (ii) subchondral bone MSC therapy achieved similar clinical scores as TKA knees in short and long-term follow ups; and (iii) subchondral bone MSC therapy could effectively reduce the size of BMLs in OA patients, and postpone or prevent the first TKA surgery in young patients with secondary osteonecrosis related to corticosteroids (Table 3, Study 3).

**Table 3:** Clinical results of subchondral bone MSC therapy for OA patients.

**Bilateral clinical study**		**OA condition before treatment**	**No. of Knees**	**Age/Gender**	**Treatment details**	**Clinical results**		**References**
						**Short-term follow-up**	**Long-term follow-up**	
1	MSC subchondral bone injection	Bilateral knee osteoarthritis	60	61 y (48–72 y) 25M/35F	5,727 BMSCs/mL; 20 mL total (10 mL in medial tibial plateau; 10 mL in medial femoral condyle)	Knee score (2 years): 79.3 ± 12; Elevated K-L score (2 years): 2/60	Yearly arthroplasty incidence: 1.3%; TKA incidence: 20% (12/60)	Hernigou et al., 2020a
	MSC IA injection		60		5,727 BMSCs/mL; 20 mL into joint	Knee score (2 years): 64 ± 21; Elevated K-L score (2 years): 11/60	Yearly arthroplasty incidence: 4.6%; TKA incidence: 70% (42/60)	
2	MSC subchondral bone injection	Planned for staged-bilateral TKA for medial osteoarthritis; “comparable” pain in both knees	140	75.4 y (65–90 y) 53M/87F	7,800 BMSCs/mL; 20 mL total (10 mL in medial tibia plateau; 10 mL in femoral condyle)	Average BML size (before treatment): 3.4 cm^3^ (0.4–6.9) Average BML size (2 years): 2.1 cm^3^ (1.2–5.7)	TKA incidence: 18% (25/140)	Hernigou et al., 2020b
	TKA		140		TKA	Average BML size: N/A	TKA revision incidence: 15% (21/140)	
3	MSC subchondral bone injection	Bilateral osteoarthritis; secondary to knee ON related to corticosteroids	30	28 y (18–40 y) 12M/18F	6,500 BMSCs/mL; 40 mL (10 mL in medial tibial plateau; 10 mL in medial femoral condyle; 10 mL in lateral tibial plateau; 10 mL in lateral femoral condyle)	Knee score (3 mo): 81.3 ± 12	TKA incidence: 10% (3/30)	Hernigou et al., 2018
	TKA		30		TKA	Knee score (3 mo): 79 ± 21	TKA revision rate: 20% (6/30); re-revision: (2/6)	

*MSCs, mesenchymal stem cells; IA, intra-articular injection; BMLs, bone marrow lesions; TKA, total knee arthroplasty; ON, osteonecrosis*.

The exact injection site and optimal delivery technique for bone marrow concentrate/MSC are of high significance for these studies. Surgeons used fluoroscopy to direct the trocar to subchondral bone marrow lesions, reaching a distance of 5 mm distal from the tidemark to avoid perforating the cartilage and to keep the calcified cartilage intact; such an approach ensures precise delivery of MSCs to the injury sites of subchondral bone in clinical practice. Delivery sites (subchondral bone *vs*. intraarticular) of MSCs are also of importance: 20% of the knees (12 in 60) in the subchondral implantation group underwent TKA at a mean of 14 years (range, 9–16 years), while 70% of the knees (42 in 60) in the intra-articular implantation group underwent TKA at a mean of 7 years (range, 3–14 years) (Hernigou et al., 2020a).

## OA Subtypes and Therapeutic Targets

OA is a heterogeneous and multifactorial disease, and the disease is described and categorized into subtypes on the basis of pathological mechanisms and clinical phenotypes. Defining the OA subtypes is clinically relevant to provide insights into the pathological process as well as to generate future therapeutic guidelines for specific patient groups. For example, Sun et al. (2018) subcategorized OA patients into two groups, pEGFR^high^ and pEGFR^low^, according to the level of expression of phosphorylated epidermal growth factor receptor (pEGFR) in cartilage (Sun et al., 2018). Inhibition of EGFR signaling pathway in murine chondrocytes by gene knockout or using the EGFR inhibitor Gefitinib was found to attenuate cartilage degeneration in mouse OA model. These findings suggest a rationale for exploration of Gefitinib as a potential intervention therapeutic to treat OA patients in the pEGFR^high^ subgroup.

Herrero-Beaumont et al. (2017) characterized four subtypes of OA, including biomechanical, osteoporotic, metabolic, and inflammatory OA. Karsdal et al. (2014) proposed six potential OA subtypes, including low-grade autoimmunity, inflammation, genetic, hormonal, metabolic and mechanotransduction subtypes, and categorized the drivers of OA into at least three different types based on the most active or affected joint tissues, including cartilage, bone and inflamed synovium. Recent work based on transcriptomic profiles of peripheral blood mononuclear cells identified two OA subtypes, a degenerative subtype, and an inflammation related subtype (Zhao et al., 2018). Presented below is a discussion of potential OA subtypes on the basis of current reported pathology and intervention studies and clinical findings on the response profile of subchondral bone remodeling.

### Profile of the OA Subchondral Bone Remodeling Responders

Four potential OA subtype are proposed: (1) responders to bone-acting agents; (2) responders to NSAIDs (Bingham et al., 2006); (3) responders to MSC-subchondral bone/regenerative therapy; and (4) responders to mechanical stimuli.

#### Responders to Bone-Acting Agents

Bone acting agents that are reported to be beneficial for OA patients include estrogen (Carbone et al., 2004), BPs (Carbone et al., 2004; Hayes et al., 2020), and SrR (Reginster et al., 2013; Bruyere et al., 2014). Effective responders to estrogen and BPs are mostly postmenopausal women at early stage of OA, and non-overweight; administration of estrogen or BPs reduced radiographic knee OA progression, and is correlated to less subchondral bone lesions (Carbone et al., 2004; Hayes et al., 2020). Responders to SrR are generally early OA patients from both genders over 50 years old with joint space width 2.5–5 mm from the SEKOIA clinical trial (ISRCTN41323372) (Reginster et al., 2013; Bruyere et al., 2014). It is noteworthy that while estrogen could prevent subchondral bone lesions, reduction of knee pain was not observed (Carbone et al., 2004); thus, using bone-acting agents for subchondral bone remodeling is more likely to be a preventive step at the very early phase of OA.

#### Responders to NSAIDs

OA patients are reported to have different types of subchondral bone osteoblast behavior, characterized by difference in endogenous production levels of PGE2 (Massicotte et al., 2002; Kwan Tat et al., 2008), i.e., low PGE2 OA [L-OA] or high PGE2 OA [H-OA] (Massicotte et al., 2002). The L-OA osteoblasts produce higher level of RANKL and exhibit a lower ratio of OPG/RANKL compared with healthy subchondral bone osteoblasts, suggesting that L-OA osteoblasts may play a causal role in subchondral bone resorption in certain subtypes of OA (Kwan Tat et al., 2008). In addition, Tu et al. (2019) identified another subchondral bone responder subtype as COX-2^+^ OA and COX-2^−^ OA in animal models, according to expression level of COX-2 in subchondral bone, and the elevated levels of COX-2 in subchondral bone accelerated cartilage degeneration at the onset of OA. These patients with PGE2^high^/COX-2^+^ subchondral bone osteoblast are highly likely to be the responders to NSAIDs.

#### Responders to MSC (Subchondral Bone Application) and Regenerative Therapy

From the patient inclusion criteria of subchondroplasty and the cell-based therapies (Table 2), it is clear that regenerative therapies are the frontline choices to bone marrow lesions (Hernigou et al., 2020a), osteochondral defects, and injuries (Brittberg et al., 1994; Wakitani et al., 2002, 2007; Kuroda et al., 2007; Peterson et al., 2010; Jiang et al., 2016).

Hernigou et al. provided a wide spectrum patient profile for MSC-based therapy for subchondral bone remodeling to prevent OA development and minimize TKA (Table 3). In the 140-case study, patients scheduled for TKA who were administrated subchondral injection of BMSCs reported good joint preservation results after 15 years of follow up (Hernigou et al., 2020b). In the young osteonecrosis patients, 96% of those who received subchondral bone MSC injection showed postponement of the first TKA for more than 10 years, while a 20% revision rate was found in the TKA treatment group (Hernigou et al., 2018). Subchondral bone MSCs treatment was found to be applicable for both early and late stage OA in terms of subchondral bone remodeling and joint preservation. These investigators also found that: (i) persistent BML larger than 3 cm^3^ after MSC therapy was an independent risk factor for TKA; and (ii) incidence rates of arthroplasty were higher for young patients and for patients with severe malalignment (hip-knee-ankle angle < 170°) (Hernigou et al., 2020b). In other words, responders to MSC subchondral bone injection were most likely individuals whose BML was no larger than 3 cm^3^ and without lower limb malalignment.

#### Responders to Mechanical Stimuli

Two obvious effective management strategies for OA related to mechanical stimuli are weight loss and exercise, both of which are recommended by the American College of Rheumatology (Kolasinski et al., 2020). A pre-clinical study showed that body weight-supported treadmill training (lower than 60% loading in the joint) is more efficient in maintaining cartilage integrity and attenuating subchondral bone loss and remodeling than treadmill training alone (Hao et al., 2020). Weight loss and exercise are therefore highly beneficial for early OA patients.

Lower limb malalignment alters the stress distribution across the knee, resulting in altered bone remodeling in tibiofemoral joint, and is a major contributor to severity of OA. Patients with malalignment are mechanical stimuli responders, and the conditions can be attenuated by correction of malalignment. The subchondral bone remodeling before and after osteotomy or other malalignment correction surgeries can be observed by bone scintigraphy, which measures reflects alterations in the metabolic activity of bone (Kraus et al., 2009), dual energy X-ray absorptiometry (Dexa), and MRI.

Knee joint distraction (KJD) is a surgical procedure involving the gradual separation of the two joint surfaces by an external fixator frame (Intema et al., 2011). Interestingly, separation and mechanical unloading of OA affected joint surfaces could reduce tissue wear and tear. Intema et al. (2011) reported a decrease in subchondral bone density on the OA affected compartment after KJD for 12 months, delaying the progression of late OA. Goh et al. (2019) recently reviewed the role of KJD in OA management and summarized available supportive evidence for the beneficial outcomes. Therefore, KJD could be a promising, clinically available therapy for OA patients, based on the reduction of mechanical stimuli to prevent OA development. In addition, exoskeletons have also shown promise in modifying the biomechanical environment of the knee in OA patients (Mcgibbon et al., 2017).

### Future Targets Under Investigation

Several new cellular and molecular targets of OA subchondral bone remodeling have been recently reported in pre-clinical animal studies, including TGFβ, PGE2/Cox2, vascularization, and mechanical sensor proteins (Figure 2). These mechanistic studies are investigated with small animals, and the targets and their possible pharmacological manipulation are summarized in Table 1. Among these, the known OA subchondral bone regulatory signaling pathways of estrogen, TGFβ, and COX2/PEG2 have been discussed in previous sections. Additional details on the regulators of the vascular and mechanical responses in OA subchondral bone are provided below.

Increased vascularization of subchondral bone and the invasion into osteochondral junction are important pathological characteristics of OA (Suri et al., 2007). The vascularization in subchondral bone is one of the potential targets. In the arthritic joint, VEGF signaling contributes to bone resorption and destruction *via* recruitment and activation osteoclasts (Matsumoto et al., 2002). Administration of bevacizumab, a neutralizing VEGF antibody, either intra-articularly (1 mL; 25 mg/mL; 3 weeks) or intravenously (5 mg/kg; 2 weeks), has been confirmed to attenuate OA in a rabbit model (Nagai et al., 2014). Su et al. (2020) discovered that preosteoclast-derived PDGF-BB enhanced the H type vessel numbers in OA subchondral bone (Kusumbe et al., 2014; Su et al., 2020). In addition, Hu et al. (2020) reported that H-type vessels and MSCs were coordinated in a positive feedback loop *via* focal adhesion kinase (FAK) signaling in MSCs, subsequently causing aberrant bone formation in ACLT-induced murine OA subchondral bone (Hu et al., 2020); furthermore, the FAK inhibitor defactinib effectively suppressed the positive loop between H type vessels and MSCs, and alleviated OA in mice (Hu et al., 2020) (Table 1).

As both cartilage and bone are mechanical sensitive joint tissues, the mechanosensitive pathway in subchondral bone represents another potential target. The expression of the mechanosensing molecule, Yes-associated protein (YAP), in human knee joint articular cartilage was positively correlated with cartilage ECM stiffness. In OA mouse cartilage, YAP expression was aberrantly increased, and conditional knockout of YAP protected against cartilage degeneration. Intra-articular administration of Verteporfin, a YAP-selective inhibitor, also lowered the expression of YAP and alleviated cartilage degeneration in DMM-induced murine OA model (Zhang et al., 2020). Wang et al. (2020) recently identified PIEZO1 as the major skeletal mechanosensor or mechanostat protein that tunes bone homeostasis. Mice deficient in Piezo1 gene expression in osteoblastic cells, while showing loss of bone mass and spontaneous fractures with increased bone resorption, are resistant to further bone loss and bone resorption due to mechanical unloading, suggesting that PIEZO1 in osteoblasts controls osteoblast-osteoclast crosstalk in response to mechanical forces. PIEZO1 regulates the YAP-dependent expression of collagen types II and IX, which in turn regulate osteoclast differentiation and bone remodeling. Additional support is provided by the findings of Zhou et al. (2020) who also reported that Piezo1/2 could regulate bone formation by mediating mechanotransduction *via* the NFAT/YAP1/ß-catenin axis in mice (Zhou et al., 2020). The manner and approach of regulating the mechano-modulatory factors and cellular activities in OA subchondral bone thus requires further exploration. Definitive classification of the nature of impairment of mechanoresponsive pathways in subchondral bone would be helpful to identify new subtypes of OA patients.

## Conclusion

In summary, we have provided a review of the key cellular and molecular players in subchondral bone remodeling, and the changes that accompany the pathogenesis of OA in the articular joint. These factors and associated pathways are potential targets for development of OA therapies focused on subchondral bone remodeling. Brief profiles of the different types of OA responders to subchondral bone remodeling are presented, based on available clinical therapies. Finally, we have also summarized the novel mediators of OA subchondral bone remodeling that are currently under investigation.

## Author Contributions

YJ: conceptual design. YC, XZ, and YJ: data mining and collection. XZ, YJ, and YC: manuscript preparation. YJ, RT, and PY: manuscript editing. PY, RT, and YJ: data interpretation. All authors contributed to the article and approved the submitted version.

## Conflict of Interest

The authors declare that the research was conducted in the absence of any commercial or financial relationships that could be construed as a potential conflict of interest.

## Abbreviations

ACLT: anterior cruciate ligament transection
ADSCs: adipose-derived mesenchymal stromal cells
ALP: alkaline phosphatase
BMLs: bone marrow lesions
BMSCs: bone marrow-derived mesenchymal stromal cells
BPs: bisphosphonates
BSMs: bone substitute materials
CaP: calcium phosphate
COL1: collagen type I
COX-2: cyclooxygenase-2
CTSK: cathepsin K
Dexa: dual energy X-ray absorptiometry
DKK2: Dickkopf-2
DMOAD: disease-modifying osteoarthritis drug
DMP1: dentin matrix acidic phosphoprotein 1
ECM: extracellular matrix
EGFR: epidermal growth factor receptor
FAK: focal adhesion kinase
HTO: high tibial osteotomy
ICOAP: intermittent and constant osteoarthritis pain
ICRS: International Cartilage Repair Society
IGF1: insulin-like growth factor 1
IKDC: International Knee Documentation Committee
KJD: knee joint distraction
KL: Kellgren-Lawrence
KOOS: Knee Injury and Osteoarthritis Outcome Score
MACI: matrix induced autologous chondrocyte implantation
MMX: medial meniscectomy
MMP: metalloproteinase
MRI: magnetic resonance imaging
MSCs: mesenchymal stem cells
mTOR: mammalian target of rapamycin
mTORC1: mechanistic target of rapamycin complex 1
OA: osteoarthritis
OCN: osteocalcin
ODF: osteoclast differentiation factor
OPG: osteoprotegerin
OPGL: osteoprotegerin ligand
ON: osteonecrosis
PBMCs: peripheral blood mononuclear cells
PDGF-BB: platelet-derived growth factor-BB
PGE2: prostaglandin E2
PLR: perilacunar/canalicular remodeling
RANK: receptor activator of nuclear factor kappa-B
PTH: parathyroid hormone
PTHrP: parathyroid hormone related peptide
RAL: Raloxifene
RANKL: receptor activator of nuclear factor kappa-B ligand
SERMs: selective estrogen receptor modulators
SMSCs: synovium-derived mesenchymal stromal cells
SrR: strontium ranelate
TGFβ1: transforming growth factor β1
TKA: total knee arthroplasty
TRAP: tartrate-resistant acid phosphatase
UC-MSCs: umbilical cord-derived mesenchymal stromal cells
VAS: visual analog scale
VEGF: vascular endothelial growth factor
WOMAC: Western Ontario and McMaster Universities Arthritis Index
YAP: Yes-associated protein.
